# Impacts of body positions on the geniohyoid muscle contraction and swallowing difficulty in healthy adults

**DOI:** 10.1002/cre2.760

**Published:** 2023-07-05

**Authors:** Fuwen Lu, Tatsuma Okazaki, Junko Okuyama, Shin‐Ichi Izumi

**Affiliations:** ^1^ Department of Physical Medicine and Rehabilitation Tohoku University Graduate School of Medicine Sendai Miyagi Japan; ^2^ Center for Dysphagia of Tohoku University Hospital Sendai Miyagi Japan; ^3^ Department of Physical Medicine and Rehabilitation Tohoku University Graduate School of Biomedical Engineering Sendai Miyagi Japan

**Keywords:** aspiration, aspiration pneumonia, body position, swallowing muscle

## Abstract

**Background:**

Body positions affect swallowing and gastroesophageal reflux. Swallowing impairment is one of the main causes of aspiration pneumonia. To prevent pneumonia, evaluation of body positions on gastroesophageal reflux recommended 30 degrees or higher semi‐recumbent positions. The geniohypoid muscle and tongue play central roles in swallowing. However, the effects of body positions on contracting rates in the geniohyoid muscle and tongue pressure are unclear. Moreover, correlations between geniohyoid muscle contracting rates and subjective swallowing difficulties are unclear.

**Aims:**

This study aimed to identify the proper body positions on contracting rates in the geniohyoid muscle, tongue pressure, and subjective swallowing difficulties.

**Materials & Methods:**

Twenty healthy adults swallowed 15‐ or 50 ml of water at 90 degrees sitting, 60‐ and 30 degrees semi‐recumbent, and 0 degrees supine positions. We scored the subjective swallowing difficulties and measured the tongue pressure and the number of swallows. An ultrasound evaluated the geniohyoid muscle size and contracting rates.

**Results:**

At sitting and 60 degrees semi‐recumbent positions, the geniohyoid muscle showed greter contracting rates than at 30 degrees semi‐recumbent and supine postions (P < 0.05), which resulted in easier swalloiwng. Greater tongue pressure was weakly correlated with fewer swallows (r = ‐0.339, P = 0.002), whereas the body positions did not affect.

**Conclusion:**

Considering swallowing and gastroesophageal reflux together, a trunk angle of 60 degrees or more might be beneficial for reducing the risk of aspiration.

## 1 INTRODUCTION

The number of deaths caused by aspiration and non‐aspiration pneumonia is increasing due aging of the population worldwide (Okubo et al., 2023; Shiokawa et al., 2023). Impairments in swallowing and cough reflexes cause aspiration pneumonia (Nihei et al., 2015; Yamaya et al., 2001). People can classify aspiration into silent or apparent aspiration, without or with evident aspiration, respectively (Teramoto, 2022). Silent aspiration is a major risk factor for pneumonia in older people, which accounted for 40%–60% of aspiration pneumonia (Ebihara et al., 2005; Ramsey et al., 2005). Older pneumonia patients often visit hospitals with nonapparent symptoms, such as anorexia and general illness. In addition, their disease condition is often severe (Ebihara, 2022). Thus, the detection of aspiration is important, and prevention is central among management strategies for aspiration pneumonia (Ebihara, 2022; Okazaki et al., 2020).

Body position affects swallowing. Swallowing requires the proper activity of the swallowing muscles, such as contraction of the geniohyoid muscle and the tongue (Feng et al., 2013; Inokuchi et al., 2016; Komatsu et al., 2018; Matsuo & Palmer, 2008). The geniohyoid muscle moves the hyoid bone and plays a central role in swallowing (Paik et al., 2008; Pearson et al., 2011). Generally, the muscles show the strongest force at the optimum length (Bolsterlee et al., 2017; Frontera & Ochala, 2015; Hunter & Faulkner, 1997). Therefore, muscle length and contraction are important for strong muscle force. Previous studies analyzed the swallowing muscles using devices such as videofluorography and electromyographic activity. However, the effects of body positions on swallowing varied between the studies (Shiino et al., 2016). The majority of the studies showed no difference between the body positions, whereas other studies showed differences such as in the duration of the swallowing and electromyographic amplitude levels in healthy adults (Barkmeier et al., 2002; Inagaki et al., 2007; Perry et al., 2012; Sakuma & Kida, 2010; Yabunaka et al., 2012). In clinical settings, semi‐recumbent positions reduced aspiration more than supine positions (Shiino et al., 2016). Recent swallowing studies often evaluate the geniohyoid muscle by ultrasound (Feng et al., 2015). Currently, the effect of body positions on its shape and contracting rate in the geniohyoid muscle is unclear.

Body position affects gastroesophageal reflux also. In mechanically ventilated patients with nasogastric tubes, the supine position increased aspiration more than the semi‐recumbent position at 45 degrees (Drakulovic et al., 1999). In older people, keeping a sitting position after meals reduced pyretic days more than a supine position (Matsui et al., 2002). The mechanisms for the above findings were suggested as minimizing gastroesophageal reflux and preventing aspiration of gastric contents into airways. Considering the risks for gastroesophageal reflux and safe swallowing, the required body angles are currently unclear.

Dysphagia was associated with low tongue pressure in older people (Maeda & Akagi, 2015). However, maximum tongue pressure was not different between sitting and Fowler's position in healthy young people (Yoshikawa et al., 2021). Currently, few studies have shown the effect of other body positions on tongue pressure.

Videofluorographic studies showed the shortening of the pharynx during swallowing. The shortening of the pharynx was induced by the contraction of the suprahyoid muscles, especially the geniohyoid muscle. This movement generates propulsive force for propelling oropharyngeal contents, characterizing a role of the contraction rates of the geniohyoid muscle (Palmer et al., 2000; Perry et al., 2012). Moreover, a computed tomography analysis showed an association between atrophy of the geniohyoid muscles and aspiration, including the possibility of silent aspiration (Feng et al., 2013).

Most previous studies evaluated swallowing objectively, such as by muscle activity. In contrast, a few studies evaluated swallowing subjectively. Therefore, information about the effects of body positions on swallowing difficulties by subjective evaluation is not sufficient. Moreover, correlations between swallowing difficulties by subjective evaluation and geniohyoid muscle contracting rates are unclear.

Previous studies reported the onset of silent aspiration during sleep or at night (Kikuchi et al., 1994; Teramoto, 2022). The mechanisms of the onset only during sleep or at night were unclear. However, considering the risk factors for the onset of aspiration pneumonia, decreased levels of consciousness and supine positions could be the possible candidates.

This study aimed to identify the effects of body positions on the contracting rates of swallowing‐related muscles and the swallowing difficulties in healthy participants. We chose the geniohyoid muscle and tongue as representative swallowing muscles to evaluate the contracting rate and muscle force, respectively. In addition, we aimed to apply this study as a preliminary step to identify the appropriate position to reduce the risk of aspiration together with the risk of gastroesophageal reflex in older people. We hypothesized greater contracting rates of the swallowing‐related muscles and easier swallowing at the body positions closer to the sitting than supine positions.

## 2 METHODS

### 2.1 Study design and participants

Twenty participants joined this study, 10 males and 10 females, 24.6 ± 1.2 (mean ± standard deviation) years old. Healthy participants aged between 20 and 65 years were recruited using online and printed advertisements. We excluded participants with a history of dysphagia, speech or respiratory disorders, and neurological, muscular, or otolaryngological trauma/disorders. The study period was between March 2021 and March 2022 and was approved by the Ethics Committee of Tohoku University Graduate School of Medicine (No. 2020‐1‐1151). Before joining the study, we explained and obtained consent from participants. This study was performed under the ethical standards in the 1964 Declaration of Helsinki.

### 2.2 Body positions

**Figure 1:**
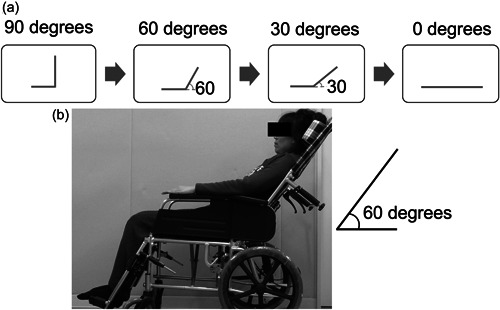
The body positions during the measurement. (a) We set 4 angles as the body positions. From left to right, 90 degrees at the sitting, 60 and 30 degrees at two semi‐recumbent positions, and 0 degrees at the supine position. (b) A wheelchair with adjustable backrest angles was applied. Participants sat in a wheelchair with their heads positioned midline and stabilized by a headset. The neck position was fixed at a slight chin‐down position. The lower limbs were bent at the knees, and the feet were on a stand.

We set four angles between the body trunk and the floor as the body positions with the femurs parallel to the floor. The angles were 90 degrees at the sitting position, 60 and 30 degrees at two semi‐recumbent positions, and 0 degrees at the supine position (Figure 1a). Participants sat in a wheelchair with adjustable backrest angles (Figure 1b). The head was positioned midline, and a headrest stabilized the head. The neck position was fixed at a slight chin‐down position with the headrest (Figure 1b). The lower limbs were bent at the knees, and the feet were on a stand (Figure 1b). We evaluated the geniohyoid muscle by ultrasound and asked participants to swallow water to evaluate their swallowing. To avoid the potential risk of aspiration and facilitate subjective evaluation by the participant, we set the order of measurements from 90 to 0 degrees.

### 2.3 Maximum tongue pressure

The maximum tongue pressure was measured by a JMS tongue measurement device (TPM‐01; JMS Co Ltd.) (Okazaki et al., 2021). The pressure was measured three times in each body position with 30 s break between each measurement, and the strongest was taken. We set the order of measurements the same as swallowing water, from 90 to 0 degrees.

### 2.4 The shapes and contracting rates of the geniohyoid muscle

**Figure 2:**
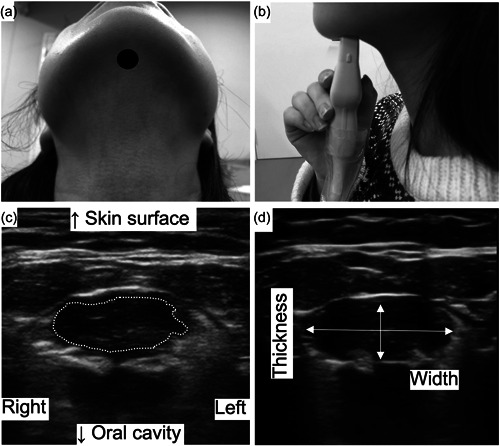
The ultrasound analysis of the geniohyoid muscle. (a) We assessed the distance between the mandible and the hyoid bone. We marked a position one‐third from the mandible as the measurement point. (b) We located a probe at the point in the coronal plane. (c) The white dotted line is the geniohyoid muscle. We positioned the probe at the marked point, traced the edge of the geniohyoid muscle, and measured the cross‐sectional area. (d) We measured the width and thickness of the geniohyoid muscle, as shown in (d). The horizontal arrow shows width, and the vertical arrow shows thickness. To avoid readers' confusion, we divided images for measurement, one for the area (c) and another for length (d).

The shapes and contracting rates of the geniohyoid muscle were recorded using an ultrasound (Viamo c100, Canon Medical Supply Co., Ltd.) and a linear probe (PLU‐704ST, Canon Medical Supply Co., Ltd.). The parameters in this study were gain 142, frequency 10.2 MHz, and depth 4.44 cm. The measurement was performed as previously shown (Feng et al., 2015). Before the measurement, the distance between the mandible and the hyoid bone was assessed. We marked a position one‐third from the mandible as the measurement point (Figure 2a). A probe was located at the point in the coronal plane (Figure 2b). We stabilized the head position with a headrest to keep the same probe position at different postures. First, participants kept 15 mL water in their oral cavity and began to swallow when instructed to start. Simultaneously, ultrasound recorded the contraction of the geniohyoid muscle during swallowing in the video. The video was played back 30 frames per second frame‐by‐frame and edited by Adobe Premiere Pro 2020 software (Adobe). The edited images were subjected to measure the cross‐sectional area (S1) of the geniohyoid muscle at rest (Figure 2c) and the maximum cross‐sectional area (S2) of the muscle during contraction, and the width (W [mm]) and thickness (T [mm]) at rest (Figure 2d) using ImageJ software. Scale bars in the edited images were applied for calibration of the length. This study set the ratio of width to thickness (W/T) as a readout of the shapes of the geniohyoid muscle. The contracting rate of the geniohyoid muscle was calculated as (S2 − S1)/S1 × 100%.

### 2.5 The scoring of swallowing difficulty

The participants were asked to swallow 50 mL of water freely at each body position to evaluate their swallowing difficulty. The swallowing difficulty is a category scale in which the participants select a number from 0 (*very easy to swallow*) to 10 (*maximum difficulty to swallow*). In addition, the number of swallows was recorded after swallowing. Finally, participants were asked to report any fear or anxiety during swallowing.

### 2.6 Statistical analysis

One‐way analysis of variance with Bonferroni post hoc analysis was used to analyze maximum tongue pressure, shapes and contracting rates of the geniohyoid muscle, subjective scoring of swallowing difficulties, and the number of swallows. The correlation between the parameters was tested by Spearman's rank correlation coefficient. Values are described as mean ± standard error. All statistical analyses used SPSS Statistics 26.0 software (IBM Corp.), and *p* values <.05 were considered significant.

## 3 RESULT

**Figure 3:**
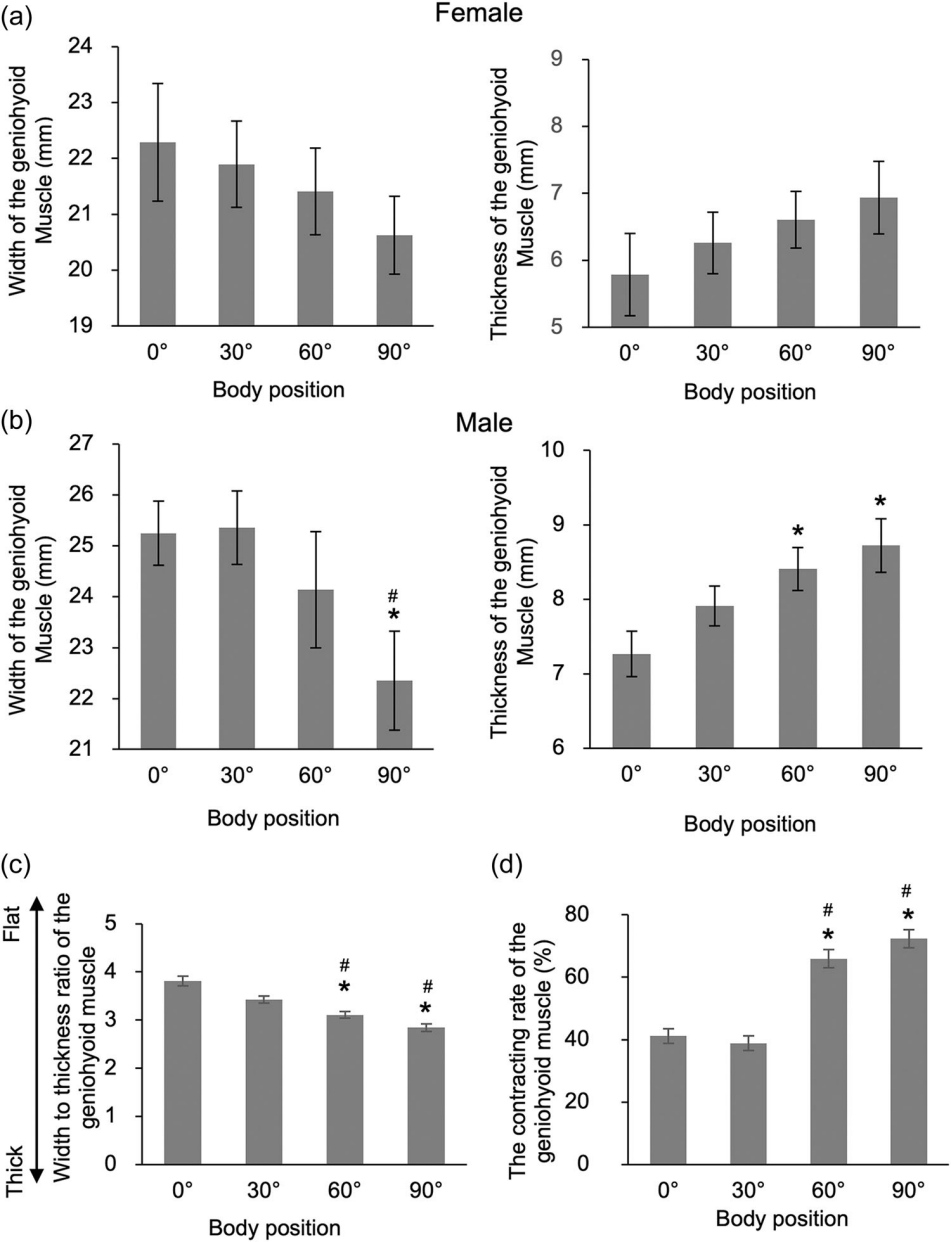
The shape and contracting rate of the geniohyoid muscle at four positions. (a) Width (left panel) and thickness (right panel) of the geniohyoid muscle at four body positions in females. (b) Width (left panel) and thickness (right panel) of the geniohyoid muscle at four body positions in males. (c) Width to thickness ratio of the geniohyoid muscle at four body positions. (d) The contracting rate of the geniohyoid muscle at four body positions. Values are mean ± standard error. *p < .05 versus 0 degrees at the supine position. ^#^ *p* < .05 versus 30 degrees at the semi‐recumbent position.

We set four angles as body positions, asked participants to swallow water, and evaluated the geniohyoid muscle by ultrasound. At every body position, mean values of width and thickness of the geniohyoid muscles were greater in males than in females. Therefore, we showed the absolute mean values of the width and thickness of females in Figure 3a and those of males in Figure 3b. The width of the geniohyoid muscle had a trend to become smaller with a greater trunk angle in both gender (Figure 3a,b). However, the width of the geniohyoid muscle was smaller at the sitting position than at the supine position only in males (*p* < .05, Figure 3b). The thickness of the geniohyoid muscle also had a trend to become greater with a greater trunk angle (Figure 3a,b). Similarly, the thickness of the geniohyoid muscle was greater at sitting and 60 degrees semi‐recumbent positions than at 30 degrees semi‐recumbent and supine positions only in males (*p* < .05, Figure 3b). The ratio of the width to thickness of the geniohyoid muscle was smaller at sitting (2.84 ± 0.07, mean ± standard error) and 60 degrees semi‐recumbent (3.11 ± 0.07) positions than at 30 degrees semi‐recumbent (3.43 ± 0.07) and supine (3.81 ± 0.10) positions (*p* < .05, Figure 3c), suggesting that the greater the trunk angle, the geniohyoid muscle became thicker. We obtained similar results in each gender also. However, the cross‐sectional areas of the geniohyoid muscles at rest were not different between the body positions. The contracting rate of the geniohyoid muscle was greater at sitting (72.33 ± 2.90%) and 60 degrees semi‐recumbent (65.88 ± 2.91%) positions than at 30 degrees semi‐recumbent (38.83 ± 2.31%) and supine (41.16 ± 2.34%) positions (*p* < .05, Figure 3d). We obtained similar results after dividing the mean values for the contracting rates by gender.

**Figure 4:**
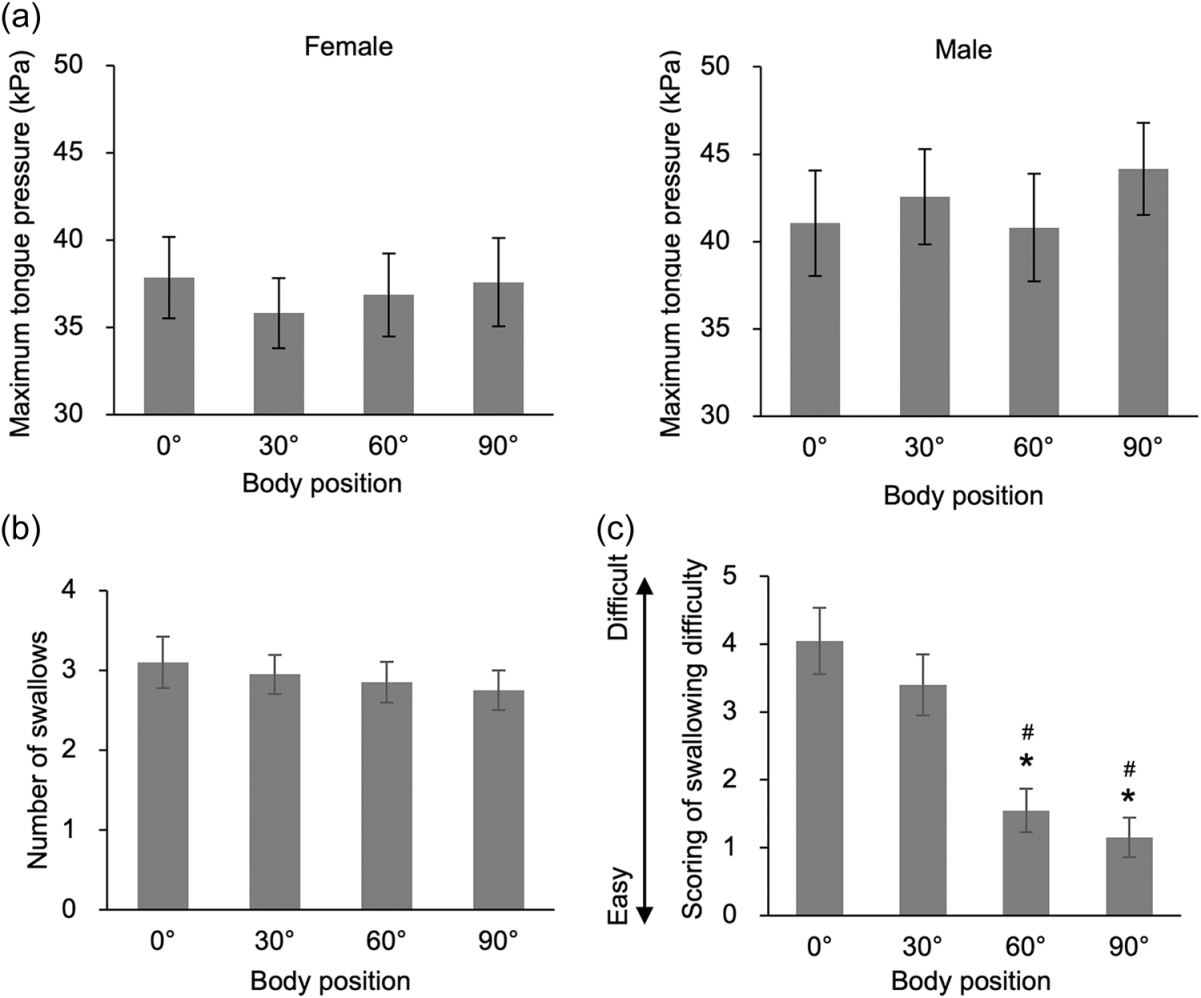
Tongue pressure, number of swallows, and subjective scoring of swallowing difficulties at four body positions. (a) Maximum tongue pressure at four body positions in females (left panel) and in males (right panel). Males showed greater tongue pressures at the sitting and 30 degrees semi‐recumbent positions than females (*p* < .05). (b) A number of swallows at four body positions. (c) Scoring of swallowing difficulties at four body positions. Values are mean ± standard error. *p < .05 versus 0 degrees at the supine position. ^#^ *p* < .05 versus 30 degrees at the semi‐recumbent position.

Tongue pressures showed a trend to be greater in males than females at every body position. Thus, we divided the tongue pressure by gender and showed females' data in a left panel and males' data in a right panel (Figure 4a). Males showed greater tongue pressures at the sitting and 30 degrees semi‐recumbent positions than females (*p* < .05, Figure 4a). However, body positions did not affect maximum tongue pressure at rest nor the number of swallows to drink 50 mL of water (Figure 4a,b). Subjective scoring of the swallowing difficulties showed easier swallowing at sitting (1.15 ± 0.29) and 60 degrees semi‐recumbent (1.55 ± 0.32) positions than at 30 degrees semi‐recumbent (3.40 ± 0.45) and supine (4.05 ± 0.49) positions (*p* < .05, Figure 4c). In addition to the difficulties, 50% of the participants felt anxiety about swallowing at 30 degrees semi‐recumbent and supine positions but not at sitting and 60 degrees semi‐recumbent positions (data not shown).

**Figure 5:**
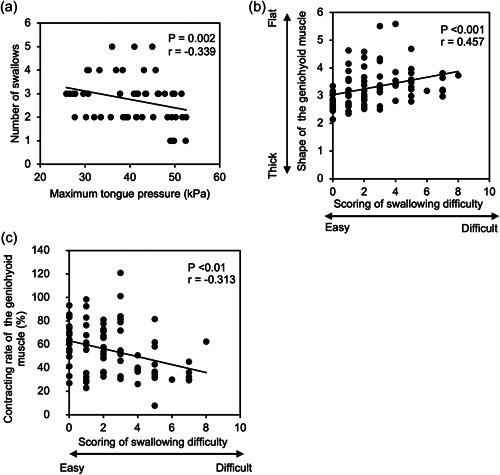
Relationships between measured indices. (a) Relationship between maximum tongue pressure and the number of swallows. (b) Relationship between subjective scoring of swallowing difficulties and shapes of the geniohyoid muscle. (c) Relationship between subjective scoring of swallowing difficulties and the geniohyoid muscle contracting rates.

Analysis of relationships between measured indices showed that maximum tongue pressure negatively and weakly correlated with the number of swallows (*r* = −.339, *p* = .002, Figure 5a). This result suggests that greater tongue pressure enables the participants to swallow more water. Scoring of the swallowing difficulties moderately correlated with the shapes of the geniohyoid muscle (*r* = .457, *p* < .001), which might suggest a possibility that positions with swallowing easiness are correlated with the thick shape of the geniohyoid muscle (Figure 5b). Scoring of the swallowing difficulties negatively and weakly correlated with the contracting rate of the geniohyoid muscle (*r* = −.313, *p* < .01), suggesting the positions with swallowing easiness are correlated with a great contracting rate of the geniohyoid muscle (Figure 5c).

## 4 DISCUSSION

In this study, we examined the effects of body positions on the objective and subjective difficulties of swallowing, such as by evaluating the contracting rates of the swallowing‐related muscles. At sitting and 60 degrees semi‐recumbent positions, the geniohyoid muscle showed greater contracting rates than at 30 degrees semi‐recumbent and supine positions, which resulted in easier swallowing. In addition, with greater tongue pressure, the number of swallows was reduced.

This study showed that participants felt easier to swallow water at 60 degrees semi‐recumbent and sitting positions than at 30 degrees semi‐recumbent and supine positions. At the supine position, the hyoid bone is closer to the trachea and moves a greater distance than at the sitting position, which was suggested as a cause of swallowing difficulty (Perry et al., 2012). When we interpret the above data as a preliminary step for application for older people, body positions at 60 degrees or more might be more advantageous to swallow than body positions at 30 degrees or less. Thus, keeping body positions at 60 degrees or more might be connected to preventing aspiration in older people.

From the point of view of the onset of pneumonia, previous studies focused on the effects of body positions on gastroesophageal reflux and subsequent aspiration of stomach contents to the lower airways (Drakulovic et al., 1999; Matsui et al., 2002). They examined the 30 and 45 degrees semi‐recumbent and sitting positions and recommended 30 degrees or higher semi‐recumbent position for 2 h after eating to prevent pneumonia (Ebihara, 2022; Fernandez‐Crehuet et al., 1997). Combined with our result for swallowing easiness, 60 degrees or higher semi‐recumbent position may have a better potential.

In this study, body positions did not affect maximum tongue pressure in healthy adults. A previous study showed a trend of greater tongue pressure at the sitting than at Fowler positions in young and older people with their heads fixed on a pillow or headrest. However, they did not show the results of statistical analysis between these positions (Yoshikawa et al., 2021). We did not examine the tongue pressure of older people, which was one of the limitations of this study.

Hyoid bone movement during swallowing is important for effectively propelling oropharyngeal contents and opening the pharyngoesophageal sphincter (Feng et al., 2015; Perry et al., 2012). One ultrasound study showed no difference in the moving distance of the hyoid bone between the body positions (Feng et al., 2015). Another study using videofluorographic analysis showed a greater anterior moving distance in the supine position than in the sitting position. However, the superior moving distance was not different, and the total moving distance was not shown (Perry et al., 2012). Thus, the effect of body position on the moving distance of hyoid bone is unclear, which we did not measure. This is one of the limitations of this study.

Recent studies showed an association between muscle weakness and pneumonia (Komatsu et al., 2018; Miyatake et al., 2022; Okazaki et al., 2020; Shiokawa et al., 2023). As for the relationship between swallowing muscles and pneumonia, a previous study showed an association between low tongue pressure and the onset of pneumonia in stroke patients (Nakamori et al., 2016). However, in general older people, there was a trend, but it was not significant (Okazaki et al., 2021). Thus, further studies are required to identify an association between the swallowing muscles and pneumonia.

Other limitations of this study. In addition to the body position, previous studies reported that head and neck positions affected swallowing (Ertekin et al., 2001; Paris‐Alemany et al., 2021; Sakuma & Kida, 2010). However, we could not change the head and neck position in this study to avoid too many settings to investigate. Next, the average age of the participants was young. In addition, technically, we could not evaluate the swallowing muscles other than the tongue and geniohyoid muscles. Thus, our future task will evaluate the effects of head and neck positions on swallowing in combination with various body positions in older people.

This study showed the effects of body positions on objective and subjective swallowing difficulties. At sitting and 60 degrees semi‐recumbent positions, swallowing was easier, and contracting rates of the geniohyoid muscle were greater than at the supine and 30 degrees semi‐recumbent positions. Considering together with gastroesophageal reflux, these results suggest that positions with a trunk angle of 60 degrees or more are recommended for healthy adults to drink water and might have the potential to prevent aspiration in older people, which is a major risk factor for pneumonia.

## AUTHOR CONTRIBUTIONS

**Fuwen Lu**: conducted the research and analyzed the data. **Tatsuma Okazaki**: interpreted the data and wrote the manuscript. **Junko Okuyama**: interpreted the data. **Shin‐Ichi Izumi**: designed the research and interpreted the data. All authors read and approved the manuscript.

## CONFLICTS OF INTEREST STATEMENT

The authors declare no conflicts of interest.

## Data Availability

The data sets generated during and/or analyzed during the current study are available from the corresponding author on reasonable request.
